# The Relationship Between Russian Kindergarteners’ Play and Executive Functions: Validating the Play Observed Behaviors Scale

**DOI:** 10.3389/fpsyg.2022.797531

**Published:** 2022-06-16

**Authors:** Aleksander Veraksa, Daria Bukhalenkova, Olga Almazova, Vera Sukhikh, Yeshe Colliver

**Affiliations:** ^1^Faculty of Psychology, Lomonosov Moscow State University, Moscow, Russia; ^2^Psychological Institute, Russian Academy of Education, Moscow, Russia; ^3^School of Education, Macquarie University, Sydney, NSW, Australia

**Keywords:** ECEC, play behaviors, executive functions, Russian teachers’ perspectives, early years

## Abstract

**Background:**

Young children’s play is theorized to develop executive functions, skills strongly predictive of many later advantages. The current study sought to validate a practicably short play behavior survey for kindergarten teachers (*N* = 18) and compare the reported behaviors to the executive functions (EFs) of their 443 Russian kindergarteners (*M*_age_ = 78.6 months; SD = 4.04).

**Research Findings:**

The factor model with satisfactory construct validity and internal consistency included three factors: leadership, play preferences and rule conformity. Analyses provide partial support for Vygotsky’s theory that play supports EF development, but particular behaviors were related to different EF components. However, kindergarteners exhibiting more leadership, preferences and conformity overall rated higher on most EF components.

**Practice and Policy:**

These findings do not support the theory that play skills improve unidirectionally with age and EFs, suggesting particular profiles of types of players and complex changes with age. The play behavior survey may be a practicable way to trace different profiles across the early years.

## The Developing Relationship Between Play and Executive Functions: A Validation Study

Executive functions (EFs) in early childhood appear to be highly significant as predictors of children’s successful school performance (Lan et al., 2011; Miller et al., 2013) and adaptation to school (Blair, 2002; Monette et al., 2011; Willoughby et al., 2012; Yeniad et al., 2013). It is therefore important to find correlates in early childhood that can be targeted through early, and preferably economical, intervention efforts (Blair and Razza, 2007; Heckman and Masterov, 2007). One prominent candidate for such efforts is play, as it has long been assumed to impact EFs (Lillard et al., 2013). Extant validated play observation tools are not practicable with large studies as they require in-depth training that cannot be easily undertaken by teachers. The current paper seeks to validate a teacher-rated scale of play behaviors to examine those that are associated with early executive functions.

### Executive Functions in the Early Years

Before children start school, inhibition and attentional control have been found to be some of the most important skills for later school success (Marzocchi et al., 2002; Blair and Razza, 2007; Duncan et al., 2007; Gilmore et al., 2013; Allan et al., 2014; Cadavid-Ruiz and del Río, 2018), and even quality of life throughout adulthood (Moffitt et al., 2011). Moreover, a broader set of skills known as executive functions (EFs)—which include working memory and cognitive flexibility as well as inhibition (Miyake et al., 2000; Karr et al., 2018)—appear to be significant predictors of children’s successful school performance (Lan et al., 2011; Miller et al., 2013; Nguyen and Duncan, 2019).

According to Miyake’s famous model of EFs, the neuropsychological basis for mastering one’s own behavior consists of a group of cognitive skills that provide targeted problem solving and adaptive behavior in new situations and came to be generally known as executive functions. They help to monitor, control thought and activities by shifting the processes toward task-related stimuli, despite the presence of secondary tasks and interference (Miyake et al., 2000). EFs are widely thought to rely on three main components: (1) inhibitory control, the suppression of a prepotent response in favor of what is required to execute the task; (2) cognitive flexibility, the ability to “shift” from one set of rules to another in order to solve problems (Huizinga et al., 2006, p. 2030); and (3) working memory, both visual and verbal, as it allows us to recall and manipulate information relevant to the task at hand (McAuley and White, 2011). Although no consensus model exists, this tripartite model has been widely validated (Garon et al., 2008; Karr et al., 2018), and, in a recent study with a demographically similar sample, provided the best fit for its data (Veraksa et al., 2020b). Despite the fact that this model was initially based on adults’ results, many researchers have applied this model to young children (Lehto et al., 2003; Diamond and Lee, 2011).

### Play in the Early Years

Play appears to be a natural activity as it has been recorded in all human societies (Lancy, 2007), and therefore conducive to broad and effective intervention efforts. Play forms the basis of early education across the vast majority of developed nations (Organization for Economic Cooperation and Development (OECD), 2017), and increasingly into the developing world (Mohite and Bhatt, 2008; Li and Chen, 2017; Utami et al., 2020). While the current study employs a theoretically consistent and culturally appropriate definition of play developed by Russian psychologist Lev Vygotsky, consistent with Russian teacher training (Slastenin, 2021), there exists ongoing international debate and contention in the English literature about how to define and identify play as a discrete behavior (Pramling Samuelsson and Carlsson, 2008; Sutton-Smith, 2009). For example, the most widely cited characteristics of play in published research in English include player enjoyment, focus on process rather than product, and freedom from external rules (e.g., Garvey, 1977/1990; Monighan Nourot et al., 1987). Yet in seeming paradox, the opposite of each of these characteristics may be as easily argued (e.g., play is freely chosen but also bound by the implicit rules of the imaginary context; Vygotsky, 2004; van Oers, 2013; Göncü and Vadeboncoeur, 2017). Interestingly, these paradoxes characterizing play revealed to Vygotsky the ways in which the child’s inhibition and other EFs are developed through play, as we elaborate in the next sections.

### Play and Executive Functions

Vygotsky’s writing pinpoints young children’s play as a key way in which children develop self-regulatory and executive capacities (Vygotsky, 1966/2016), particularly in early childhood when he theorized that play leads cognitive development (Karpov, 2005; Veraksa and Veraksa, 2021). Vygotsky defined play by the existence of an imaginary situation implied by social rules (Winther-Lindqvist, 2019). Vygotsky (1966/2016) upheld that children play to enact desires (e.g., to cook hot food) but must simultaneously regulate other impulses in order to maintain the rules implied by the imaginary situation (e.g., not running when carrying an imaginary soup). Thus the first of several paradoxes emerges: though children play in order to realize certain desires, they inadvertently must inhibit other demands to maintain the imaginary context (Vygotsky, 1966/2016). This would suggest that children practice inhibiting pre-potent responses to reality (e.g., an empty bowl) in favor of responses appropriate for the imagined (e.g., holding it horizontally and carefully) and conforming to abstract rules. The paradox also suggests children practice submitting their own desires (e.g., to carry the empty bowl most conveniently, by the rim) to the preferences of the group (i.e., as if it were easily spilt). Finally, it suggests that the child must limit their proposals of new ideas (e.g., making a sign for the restaurant) to what s/he imagines to be the interests of the group (e.g., cooking-related themes). Thus, there appear to be social and emotional as well as cognitive components of EFs that may be practiced during pretend play, constituting all EF components (see next section): cognitive flexibility (switching from imagined to real activity, imagining others’ desires and cognitive states), inhibition (restraining one’s real desires in favor of those of the role enacted), and working memory (in order to remember the motives of others, or to maintain consistent play rules and narrative).

Contemporary longitudinal and experimental evidence provides some support for the theory that EFs are developed during play. Increased free time is associated with self-directed executive functioning (Barker et al., 2014), positive adaptive behavior, and even academic success (Lehrer et al., 2014), suggesting that children’s autonomy over their activities may practice EFs (Barker and Munakata, 2015). Large longitudinal data suggest that time spent playing at 2–5 years of age predicts self-regulation, including inhibition and other EFs, 2 years later (Colliver et al., 2022). Particular EFs, such as attention shifting (Pierucci et al., 2013; White and Carlson, 2016), working memory (Thibodeau et al., 2016), delay skills (Carlson et al., 2014), and inhibitory control (Goble and Pianta, 2017; White et al., 2021; White and Carlson, 2021), have all been associated with pretend play behaviors. While studies have not conclusively shown a causal link between play and EFs (Lillard et al., 2013), the number of experimental studies linking them have increased substantially in the last decade.

### Observing Play in the Classroom

Several play behavior observation scales exist, but none appear to be practicable for large studies as they require extensive observer training to be implemented.

For example, the Child-Initiated Pretend Play Assessment (ChiPPA) is an observation-based method designed to assess a child’s solitary play in specially created conditions (McAloney and Stagnitti, 2009). The child is offered toys with clear functionality (e.g., miniature animal figures) following unstructured materials such as pieces of fabric and sticks. The level of complexity and self-organization in the play are examined by an observer for 30 min. For larger studies requiring an assessment of play behaviors, the time-consuming nature of test administration (30 min per child plus training) makes ChiPPA impracticable.

Alternatively, the Preschool Play Behavior Scale (PPBS) was designed to assess the multiple forms of young children’s solitary and social free play behaviors (Coplan and Rubin, 2001). It is a teacher rating scale that assess the three distinct forms of nonsocial (i.e., reticent, solitary-passive, and solitary-active) and social play behaviors (social and rough play). Unfortunately, this scale does not examine how well children are able to devise, determine or adhere to rules of play, as was a requirement for the specific theoretical underpinning outlined previously on play and EFs.

The Penn Interactive Peer Play Scale (PIPPS) is a questionnaire for teachers focused on interpersonal interaction in a child’s free play (Fantuzzo et al., 1995). The three factors emerging from the PIPPS, play disruption, disconnection and peer interaction, correlated with factors on an existing scale of social skills (self-control, interpersonal skills, and verbal assertion) and problem behaviors (internalizing and externalizing), suggesting its usefulness for understanding social and emotional skills. While this scale was closest to the theoretical rationale outlined earlier, the burden for each teacher to evaluate a whole class on 36 items was considered excessive for the current purpose of identifying a scale that would be practicable for large studies typically undertaken by the authors.

Two other tools for assessment have relatively similar structure: The Play Observation Scale (POS) by Rubin (2008) and The Play Observation Form (POF) by Frost (1992). Both of them represent a form of structured observation and analysis of the play components in order to classify play episodes to one of the proposed play categories. Based on different play classifications the POS and the POF create diverse combinations of play types. POF allows observers to code behaviors as either a form of cognitive play, other play, or non-play. The assessment by the POS encompasses several steps: an observed episode is first classified as play or non-play and further an assessor determines cognitive (functional, creative, dramatic (acting), rule-based) and social levels (single, parallel, or group) of play. Both tools help identify a child’s level of cognitive development through play. Training required for the use of such scales would not be practicable in the Russian context, where teachers are already significantly burdened by administrative tasks related to each child’s development and learning.

In response to the fact that most of the tools are specialized on controlled indoor environments, Loebach and Cox (2020) proposed the Tool for Observing Play Outdoors (TOPO). TOPO is an observational protocol aimed at evaluation of both children’s behaviors in outdoor environments and the play environment itself. The procedure includes observation of a child’s behavior and subsequent classification of the play episode to two of the play types which best capture the essence of the play episode. Given that outdoor activities are less constrained, TOPO introduces expanded play taxonomy including bio play, restorative and digital play.

Despite the considerable number of evaluation tools, those are problematic to apply in Russian ECEC settings. First of all, their design and content are often determined by a specific aim of the scale (e.g., PIPPS targets mainly interpersonal interactions, TOPO the environmental play interactions). The current study required a scale that could assess behaviors related to Vygotskian descriptions of play and EF development.

Secondly, the theoretical background of the scales were not culturally appropriate (e.g., ChiPPA was designed in the context of occupational therapy as guidance on further intervention plans), therefore narrowing the focus to solitary play. Current items were based on cultural-historical theory, focusing on external as well as internal functions, which fits with Vygotsky’s holistic approach to understanding development (Hedegaard, 2012). Furthermore, most teachers are acquainted with this theory, which makes the items most culturally appropriate for large scale Russian studies.

Finally, some assessment tools often require the assessors to be trained and some methods require specific setting, materials and toys for a child’s interaction to be examined (i.e., ChiPPA). Our task was to elaborate questionnaire, the use of which did not demand special additional education from teachers and which usage would take not more than 5 min for each child’s assessment.

### The Context of This Study

Extending on the work of Vygotsky (1983), the traditional Russian kindergarten tradition assumes its main educational task is to facilitate children’s acquisition of different cultural forms of knowledge and cultural tools for operating with them (symbols, models, schemes, etc.; Veraksa et al., 2010). Unlike his continental European contemporaries, Vygotsky (1997, p. 95) argued that the most significant path for the child’s cultural development was through imitation. In imitation, the adult is the bearer of the ideal forms of culture, and this preeminence surfaces in Russia’s education system from a young age (Veraksa et al., 2021). The highly disciplined Russian kindergarten pedagogy is oriented toward social norms and rules, foregrounding adult-led group work involving imitation of adults’ actions as the main avenue for knowledge acquisition (Zaporozhets, 1985). Children practice imitating the teacher and other adults in playing with each other.

Peer play occurs mostly in rest times between-lesson breaks and lunch, where it is likely to be led by children rather than teachers, who take an observational role (Yakshina et al., 2020). Kindergarteners’ peer communication takes place mainly in the process of playing together. In peer play, children do not need to follow external rules imposed by the teacher, but they need to take into account the desires and actions of another child, defend their point of view, build and implement joint plans (Smirnova and Riabkova, 2016). It is thought that peer play leads to children’s social skills and EF development. Therefore, we asked teachers mainly about free role-playing with peers.

While not all teachers have the opportunity to undertake special training to assess the complexity of each child’s play, they can assess each child’s (1) propensity to lead play, (2) how much they follow the rules, and (3) if they have any preferences for play topics (whether children are more focused on playing with peers or by themselves; for free play with peers or according to predetermined rules). These three components are also linked to Vygotsky’s theorization of the mechanisms behind play facilitating EF development, which we hypothesized as: (1) the child’s ability to initiate play with others may indicate greater cognitive flexibility in tailoring their own initiatives towards others’ interests; (2) a narrower range of play topic preferences may indicate lower cognitive flexibility as children play with less diverse content and playmates; and (3) a child’s ability to conform to the rules is likely indicative of her/his working memory and inhibition.

The current study sought to assess whether a short survey of teachers’ perspectives on children’s play behaviors is reliable and internally consistent, and whether the perspectives reflect different components of children’s EFs. The research questions were therefore:

1. Is the Play Observed Behaviours Scale (POBS) reliable and validated?
2. What are the relationships between kindergarteners’ play behaviors and EF components?

## Materials and Methods

### Participants

The study involved 443 children (51% girls) aged 6–7 years (*M* = 78.6 months; SD = 4.04) who attended 18 preparatory groups from 11 public kindergartens in Moscow, Russia. All parents were informed about the aims of the study and gave written consent for children’s involvement in the research. Considering that the whole number of preschoolers in Russia is 7.6 million, the sample of 384 children is representative according to the sociological calculator. Our study involved 434 preschoolers (>384).

In Russia, children attend kindergarten the year before elementary school entry at age 7 years. The study selected kindergartens in the districts characterized by the same level of infrastructure and designed to accommodate primarily medium-income families in the catchment area (Mean annual household income = $US 490 or 35,361 rub) and all families of the participating children identified as Russian-born. This provided a relatively homogeneous socioeconomic sample. Eighteen teachers (100% female, *M*_age_ = 45.1, SD = 10.7 years) were interviewed regarding children’s play behaviors.

All parents were informed about the aims of the study and gave written consent for children’s involvement in the research. Participants were informed that they could withdraw participation anytime without consequence and informed consent was elicited at the start of the teacher survey. All teachers were qualified teachers with 7–11 year tertiary/college degree, and with 3–30 years experience teaching.

### Measures

#### Play Observed Behaviors Scale

The focus on observable play behaviors outlined earlier in Vygotsky’s description of how play leads to development—play leadership, preferences and rule conformity—were used to generate items for the teacher survey. The resultant 8-item Play Observed Behaviors Scale (POBS) asked ECEC teachers to assess the degree to which each kindergartener in their class generally fits the play behaviors descriptions across a 3–point, Likert-type scale (1—Never expressed; 2—Occasionally expressed; or 3—Frequently expressed).

As the survey was administered by pencil and paper at the end of the kindergarten year, teachers’ answers were based on at least one full year of working with and knowing the child. Similar rating scales are frequently used for the monitoring of children’s attainment of the educational program learning outcomes, so teachers were expected to be familiar with the POBS format.

Though the survey was administered in Russian, the second author translated the questions to English:

1. The child leads peers in play;
2. Peers gladly include the child in their play;
3. The child conflicts with peers during play;
4. The child understands and follows the rules of play;
5. The child makes sure peers comply with the rules of play;
6. The child likes to engage in games with explicit rules in free time (e.g., board games, hide and seek, tag);
7. The child likes to engage in quiet projects or activities in free time (e.g., drawing, making models, building with blocks, reading, etc.)
8. The child likes to play/act out stories in free time (e.g., from real life, cartoons, films, books, etc.).

#### Executive Functions Tests

Consistent with the three factors identified in previous research (Veraksa et al., 2020b), the following five measures were used for the assessment of all three components of the executive functions. The reliability of the tools used to assess executive functions has been tested on more than 1,300 preschoolers from Russia (Veraksa et al., 2020a).

##### Cognitive Flexibility

The Dimensional Change Card Sort (DCCS; Zelazo, 2006) was used to assess the kindergartners’ cognitive flexibility. DCCS consists of three tasks for sorting cards: at first a child arranges the cards by color, then by shape, and then in accordance with a complex rule (if the card has a frame, then he/she must sort it by color, and if there is no frame, then he/she has to sort it by form). For each correctly sorted card, a child is awarded one point, at the end the number of points for each try is calculated (DCCS Sum).

##### Cognitive Inhibition

The “Inhibition” subtest of the NEPSY-II was used for assessment of cognitive inhibition. The subtest consists of two blocks: a series of white and black figures (circles and squares) and a series of arrows with different directions (up and down). Two tasks were carried out with each series of pictures: the task of naming figures (in this case a child simply had to name the figures that s/he saw as quickly as possible) and the task for inhibition (in this case a child was supposed to say the opposite of what they saw; for example, if s/he saw a square, s/he had to say “circle,” etc.). In each task, the number of errors corrected and uncorrected by the child and the amount of time spent on the task was recorded. Then the number of corrected and uncorrected errors and time were translated into a combine score using tables from the NEPSY-II manual.

##### Physical Inhibition: Statue

The “Statue” subtest of the NEPSY-II is aimed at inhibition and self-control of bodily movements. In this task, a child needs to stand motionless in a certain position for 75 s, without being distracted by external sound stimuli. For each 5-s interval the three types of mistakes made are recorded (i.e., movements, the opening of the eyes, and vocalizations) and child receives points from 0 to 2 for the successful completion of the task (maximum 30 points): child received 2 points if did not make a mistakes during 5-s interval, 1 points—if child makes one type of mistake, 0 points—if child makes 2 or more types of mistakes.

##### Verbal Working Memory

To study verbal working memory, the “Sentences Repetition” (SR) subtest of the NEPSY-II was used, translated into Russian. The subtest consists of 17 sentences, which are presented in gradually increasing difficulty (in the length and grammatical structure of the sentences) with the task to remember and repeat them. For each correctly repeated sentence, a child receives 2 points, if he/she made 1–2 mistakes while repeating (e.g., such as skipping, replacing or adding words, changing the order of words), if however a child makes 3 or more mistakes or does not answer at all then he/she is awarded 0 points.

##### Visual Working memory

To study the visual working memory and the visual–spatial orientation, the NEPSY-II “Memory for Designs” (MfD) subtest was used. In this method the following final scores are recorded: Content, which reflects the correctness of the image details’ remembering (maximum 46 points); Spatial, which reflects the correctness of the configuration’s remembering by a child (maximum 24 points); and Bonus, which is granted to the child for the correct memorization and accounting of both parameters at the same time (maximum 46 points). All three indicators are summarized into the final score (MfD Total; maximum 116 points).

##### Nonverbal Intelligence

As an additional control measures of children development, we used a nonverbal intelligence level. Non-verbal fluid intelligence was assessed with the Raven’s Colored Progressive Matrices test (Raven et al., 1998). The task included three sets of matrices, 12 items per set. Each item presented a pattern of geometric designs with a missing piece. The task was to pick the missing piece from six available options. Children were tested individually with no time limit, but the procedure was stopped when the child responded incorrectly on four items in a row. Accuracy scores were calculated (max = 36).

#### Procedure

All the tasks were performed in the second half of the school year during two individual meetings with each child (each lasting 20–25 min) in a quiet room of a child’s kindergarten. The order of the tasks was the same for each child. Using the Raven’s Colored Progressive Matrices test excluded preschoolers with suspected cognitive development disorders (three preschoolers) from the study.

A survey of teachers also took place in kindergarten: the tester received their verbal consent to fill out the questionnaire and then the teacher filled it out most often in the presence of the tester.

The study was approved by the Ethics Committee of the Faculty of Psychology at Lomonosov Moscow State University (the approval No: 219/27).

#### Analytic Procedure

After descriptive statistics for each of the measures were calculated, the Cronbach’s alpha was used as a measure of internal consistency. Сhildren in the sample (*n* = 434) were randomly split up into two sub-samples. The Exploratory Factor Analysis (EFA) was conducted with the first sub-sample (*n* = 227), then the Confirmatory Factor Analysis (CFA) on the second half of the sample (*n* = 216) was used to compute factor model fit indices. A correlation matrix revealed age correlated with each of the factors. A one-way analysis of variance (ANOVA) was conducted to identify any effect age group and sex may have had on any of the model factors. A correlation analysis was conducted to show associations between the three factors and EF components. Cluster analyses of the factors were conducted to separate groups of children based on their play behaviors.

## Results

Table 1 presents the descriptive statistics of each of the items in the initial survey and EF measures.

**Table 1:** Descriptive statistics of Play Observed Behaviors Scale (POBS) items and EF measures.

Item	*N*	Mean	SD
Play survey Q1	434	1.05	0.729
Play survey Q2	436	1.43	0.613
Play survey Q3	438	0.61	0.698
Play survey Q4	437	1.55	0.576
Play survey Q5	438	1.28	0.681
Play survey Q6	437	1.55	0.570
Play survey Q7	438	1.41	0.653
Play survey Q8	438	1.30	0.665
Cognitive flexibility	441	20.89	2.642
Cognitive inhibition	401	10.69	3.147
Physical inhibition	439	22.97	5.575
Verbal working memory	442	21.46	4.562
Visual working memory	432	84.44	22.545

Cognitive Flexibility, Dimensional Change Card Sort Total score; Cognitive inhibition, NEPSY-II “Inhibition” subtest; Physical Inhibition, NEPSY-II “Statue” subtest; Verbal Working Memory, NEPSY “Sentence Repetition” subtest; Visual Working Memory, NEPSY-II “Memory for Designs” subtest.

The Cronbach’s alpha for POBS items 0.764, which indicates acceptable reliability of the questionnaire.

First, the EFA was conducted with the first sub-sample (*n* = 227). In our exploratory model, Q2 appeared to be a part of two factors (one and three) leading us to elect a model excluding Q2. Next, we conducted an EFA, and the Kaiser–Meyer–Olkin value was 0.721 and the Bartlett’s test of sphericity approximately, *χ*^2^ = 298.741 (df = 21, *p* < 0.001), indicating that the data were suitable for factor analysis. Principal Component Analysis with Varimax method yielded a three-factor model for the scale, which accounted for 68.48% of the total variation. No cross loadings were above 0.44, as shown in Table 2.

**Table 2:** Factor loadings for three factor model.

Item	Factor loading		
	Factor 1 (Leadership)	Factor 2 (Preferences)	Factor 3 (Conformity)
Q1 Leads peers in play	**0.868**		
Q5 Makes sure peers comply with the rules of play	**0.768**		
Q6 Likes to engage in games with explicit rules		**0.536**	
Q7 Likes to engage in quiet projects or activities		**0.878**	
Q8 Likes to play/act out stories		**0.625**	
Q3 Conflicts with peers during play			**−0.877**
Q4 Understands and follows the rules of play			**0.751**
Eigenvalue	1.76	1.61	1.51
Explained variance	25.13%	22.25%	21.10%

There were no cross loadings above 0.44. Bolded values indicate loadings over 0.44 and below −0.44.

**Figure 1:**
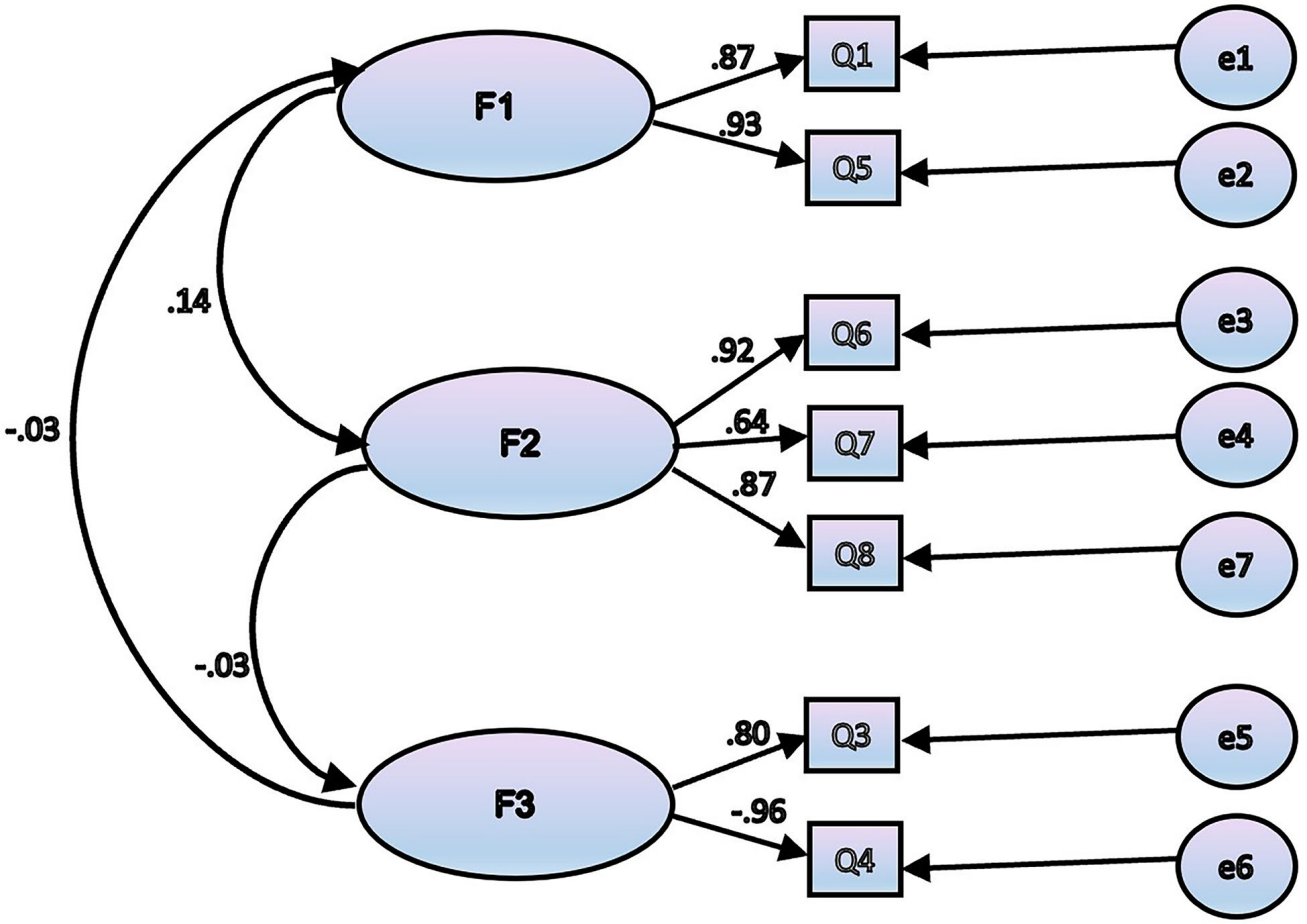
Confirmatory factor analysis model.

Second, the CFA was used with the second sub-sample (*n* = 216) to test the factor structure explored by the EFA as above. Results of CFA (Maximum Likelihood) for the three factor model (see the Figure 1) showed that the model fit was satisfactory (*χ*^2^ = 2641.71, *df* = 105, *p* = 0.000; RMSEA = 0.081; CFI = 0.931; TLI = 0.926; SRMR = 0.073).

RMSEA is the square root of the mean square of the approximation error. An RMSEA value of no more than 0.080 is considered acceptable. According to this index, the value of our model is 0.081, which is close to acceptable.

Each of the Goodness-of-Fit indices exceeding 0.9 indicate an acceptable correspondence of the model to the data. With Normed fit index (NFI) = 0.929, Tacker-Lewis index (TLI) = 0.926, Comparative fit index (CFI) = 0.931, suggest the conditions are met.

### Analysis of Factors With Age and Sex

**Figure 2:**
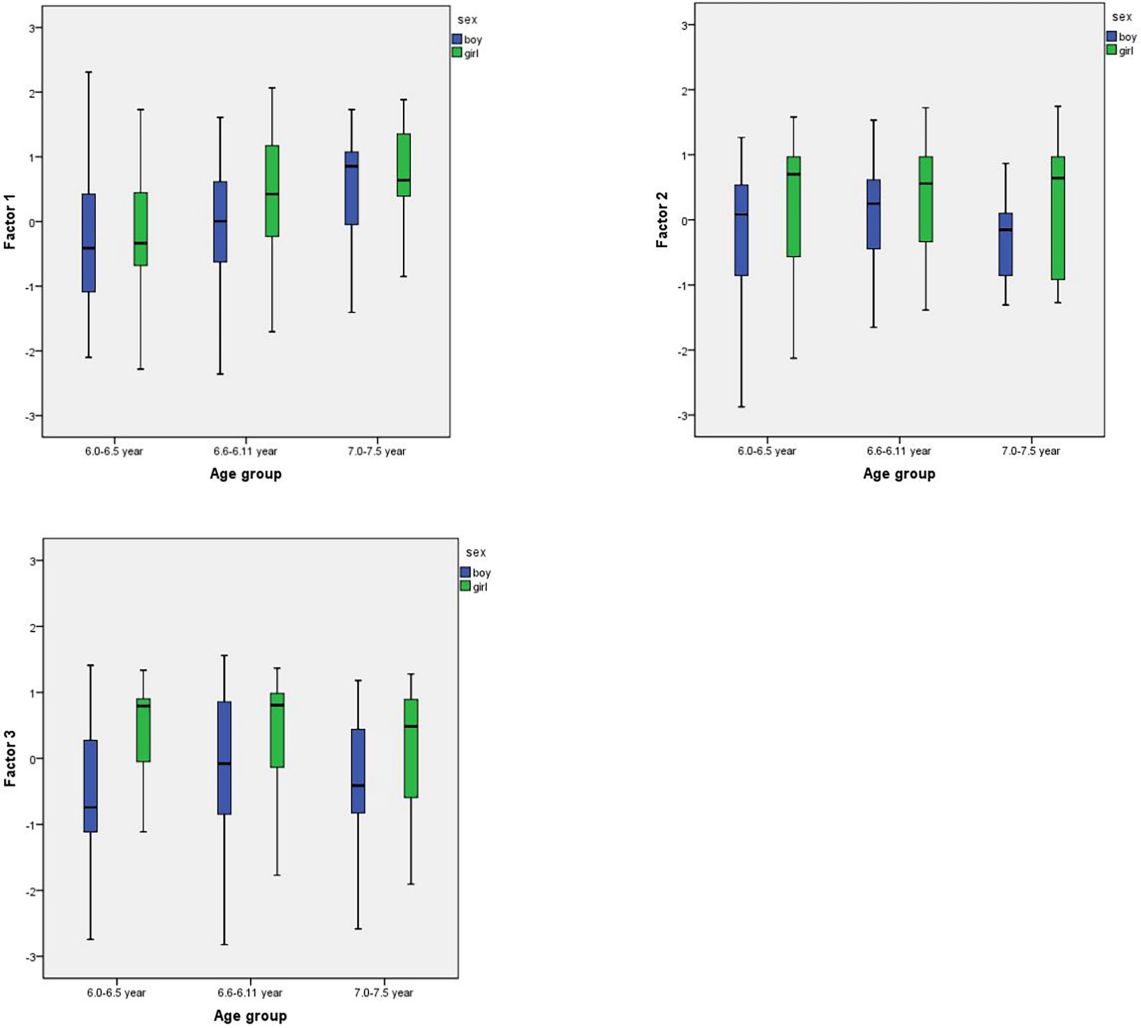
Results of sex and age analysis (Boxploys) on Factor 1 (Leadership), Factor 2 (Preferences), and Factor 3 (Conformity).

We divided sample into three age groups: 6.0–6.5 years (165 children), 6.6–6.11 years (189 children), and 7.0–7.5 years (47 children). Then we used ANOVA to analyze factors with age and sex (Table 3 and Figure 2).

**Table 3:** ANOVA of age group and sex on each of the three factors.

	df	Factor 1 (Leadership)		Factor 2 (Preferences)		Factor 3 (Conformity)	
		*F*	*p*-Value	*F*	*p*-Value	*F*	*p*-Value
Age	2	11.727	**≤0.001**	0.575	0.563	2.230	0.109
Sex	1	5.651	**0.018**	16.282	**≤0.001**	24.012	**≤0.001**

Bolded values indicate p < 0.05.

According the results of Factor 1 (Leadership) with sex and age analysis, girls have higher results (or teachers tends to assess them higher) and we can see age differences (the older the child, the higher results).

According to results of Factor 2 (Preferences) and Factor 3 (Conformity) with sex and age analysis, girls have higher results then boys in all age groups. It is interesting to note that child engagement in different types of play decreases after 7 years.

### Analysis of Relationships Between Factors and EF Components

At the first step, we performed a correlation analysis between the cumulative scores of EF tasks and each factor (see Table 4). Factor 1 (Leadership) and Factor 3 (Conformity), but not Factor 2 (Preferences), were significantly correlated with Cognitive Flexibility. Only Factor 3 was correlated with Cognitive Inhibition, but all three were correlated with Physical Inhibition. Factor 1 and Factor 3, but not Factor 2, were significantly correlated with Verbal Working Memory, whereas only Factor 3 was correlated with Visual Working Memory.

**Table 4:** Correlations between executive function components and factors.

	Factor 1 (Leadership)		Factor 2 (Preferences)		Factor 3 (Conformity)	
	*R*	*p*-Value	*R*	*p*-Value	*R*	*p*-Value
Cognitive Flexibility	0.179^**^	≤0.001	−0.018	0.713	0.125^**^	0.010
Cognitive Inhibition	0.050	0.323	0.064	0.208	0.121^*^	0.017
Physical Inhibition	0.105^*^	0.030	−0.113^*^	0.019	0.096^*^	0.048
Verbal WM	0.173^**^	≤0.001	0.074	0.125	0.203^**^	≤0.001
Visual WM	0.014	0.770	0.026	0.593	−0.105^*^	0.030

Cognitive Flexibility, Dimensional Change Card Sort Total score; Cognitive inhibition, NEPSY-II “Inhibition” subtest; Physical Inhibition, NEPSY-II “Statue” subtest; Verbal Working Memory (WM), NEPSY “Sentence Repetition” subtest; Visual WM, NEPSY-II “Memory for Designs” subtest.

* *p* < 0.05 (2-tailed);

** *p* < 0.01 (2-tailed).

Given that each EF component appeared to be significantly correlated with one or more factors, a cluster analysis (K-means method) was conducted, revealing three groups of children with different levels of ratings on their play behavior (see Table 5): the first group had the highest results in all factors (leadership, play preferences, conformity), while the second group had the lowest. These first and second cluster groups were significantly different to each other on all measures of EF (*p*’s = 0.000–0.046) except Visual Working Memory (*p* = 0.364). Results of a multiple comparison (Sheffe criterion) indicated that the first cluster scored significantly higher than Cluster 2 in all EF components except for Visual Working Memory (see Table 6).

**Table 5:** Final cluster centers.

	Cluster		
	1 (High)	2 (Low)	3 (Medium)
Factor 1 (Leadership)	0.67434	−0.17197	−0.95786
Factor 2 (Preferences)	0.38895	−0.02831	−0.67260
Factor 3 (Conformity)	0.57142	−1.06868	0.75251
Number of children	176	162	95

**Table 6:** Differences in scores of different aspects of executive functions in different clusters.

	Sum of squares	df	Mean square	*F*	Sig.	*Post-hoc* mean difference
*Cognitive flexibility*						
Between groups	77.086	2	38.543	5.630	0.004	Cluster 1 > 2
Within groups	2,923.204	427	6.846			
Total	3,000.291	429				
*Cognitive inhibition*						
Between groups	61.128	2	30.564	3.107	0.046	Cluster 1 > 2
Within groups	3,816.862	388	9.837			
Total	3,877.990	390				
*Physical inhibition*						
Between groups	201.346	2	100.673	3.366	0.035	Cluster 1 > 2
Within groups	12,709.726	425	29.905			
Total	12,911.072	427				
*Verbal WM*						
Between groups	421.016	2	210.508	10.647	0.000	Cluster 1 > 2
Within groups	8,462.246	428	19.772			
Total	8,883.262	430				
*Visual WM*						
Between groups	1,037.632	2	518.816	1.014	0.364	
Within groups	213,810.624	418	511.509			
Total	214,848.257	420				

Cognitive Flexibility, Dimensional Change Card Sort Total score; Cognitive inhibition, NEPSY-II “Inhibition” subtest; Physical Inhibition, NEPSY-II “Statue” subtest; Verbal Working Memory (WM), NEPSY “Sentence Repetition” subtest; Visual WM, NEPSY-II “Memory for Designs” subtest.

## Discussion

This study sought to develop and validate a practicable scale to measure children’s play behaviors most related to EF components. Our EFA and CFA results suggested the BOPS is reliable and validated, suggesting its applicability in kindergarten settings such as those in Russia where ECEC teachers are already burdened by administrative tasks, have a large number of children in the classroom, rigid fixed daily routine (Almazova et al., 2019) and as such a very brief scale is a practicable solution for assessing a large number of children’s play behaviors. In this regard, BOPS may be a useful addition to the range of existing scales which may be more burdensome or less culturally appropriate. For example, the Child-Initiated Pretend Play Assessment (ChiPPA), takes 18–30 min per observational session and it is administered one-on-one in a location free from distractions such as excessive noise or other children (McAloney and Stagnitti, 2009). Similarly, a tool designed for diagnostic purposes for therapists and requiring special training, the Penn Interactive Peer Play Scale (PIPPS), focuses only interpersonal interactions (Fantuzzo et al., 1995). Moreover, teachers have accumulated a greater knowledge of children in their class than would a trained observer coding videos of children’s play, as was used for the development of some play observation scales such as the PIPPS (Fantuzzo et al., 1995). Other tools assess only solitary play [Preschool Play Behavior Scale (PPBS)] or use taxonomy of play types that Russian teachers are not familiar with (e.g., Hughes, 1996) and would therefore not be culturally appropriate.

Results of the CFA suggested three types of play behaviors that were unrelated (e.g., Figure 1 shows F2 and F3 were negatively related at −0.03), which in our opinion once again confirms the correctness of using exactly such a structure to describe the questionnaire data. If we look at the semantic orientation of the issues included in different factors, then the independence of the factors becomes less unexpected. Factor 1 (Leadership) tells about how a child can manage and control other children behavior during play, Factor 2 (Preferences) about the preferred types of play activity, Factor 3 (Comformity)—about how the child himself can follow the rules. Managing one’s own and others’ behavior for preschool children may represent independent abilities. We suggesting that simple calculations of play (e.g., time spent in free play; Colliver et al., 2022) may be insufficient to accurately catalog the impact of different play behaviors on executive functions. For a more detailed study of play in subsequent studies, it will be necessary either to expand the questionnaire or use additional techniques (for example ChiPPA).

This study also sought to examine the relationships between play behaviors and EF components given the theoretical rationale for the educational provision of play in kindergarten elaborated by the cultural-historical model of child development (Vygotsky, 1966/2016; Karpov, 2005). In Russian kindergartens, child-initiated peer communication takes place mainly in the process of playing together. It is thought that in peer play, children begin to take into account the desires and actions of another child, defend their point of view, build and implement joint plans (Smirnova and Riabkova, 2016). It is therefore thought in cultural-historical theory that play is the leading, or most contributive, activity for social skills and EF development in this age (Vygotsky, 1966/2016). However, the individual characteristics of the play of different children vary significantly; for many, the play remains extremely primitive, and for some, this activity is generally supplanted by other activities (Smirnova and Riabkova, 2016). Thus, this study sought to distinguish different levels of play development *via* different play behaviors. Current results supported the notion that play leadership increased with age, while preferences and conformity appeared to drop after the child’s seventh birthday (Figure 2). Girls remained consistently higher in play behavior ratings.

One of the main play components is theorized to be the child’s acceptance of various roles (Vygotsky, 1966/2016; Karpov, 2005). Assuming a role, a kindergartener follows the rules of the role but can also exit from the role at any time, i.e., change the play situation into a real one. Thus, play activity is likely to involves all EF components: cognitive flexibility (switching from one role to another, from play activity to real activity), inhibition (use of play substitution, that is, restraining the child must limit her/his affective responses to a play situation as s/he would for a real one), working memory (used when maintaining the play rules, etc.). Cognitive flexibility was correlated with Factor 1 and 3 but not Factor 2, suggesting that children who tend to play in fixed ways or within limited topics may have fewer opportunities to practice flexibility. However, significant correlations with the statue task suggest these children may be practicing behavioral inhibition in their play regardless. Conformity with others’ rules, on the other hand, was associated with all EF components, suggesting its importance as a play behavior across all ages and both sexes. Leadership of play held associations with all EF components except cognitive inhibition and visual working memory, which may suggest a greater degree of impulsivity among children who show more initiative and leadership in play.

Our expectations about whether the scale is indicative of executive functions were partly supported by the current data, but reveal a more complex picture than simply all play behaviors being related to all EF components across the kindergarten years. The results show the first cluster of children (highest in play leadership and preferences) outperformed the second (low preferences and conformity) on almost all EF components, which suggests children might inhibit their own personal desires to maintain the imaginary context (Vygotsky, 1966/2016), because they may use attentional switching and working memory of what the context is, as well as inhibition of motives that are not relevant to the imaginary context. Similarly, the cognitive flexibility Vygotsky hypothesized necessary to play with others’ ideas and motives is consistent with the first cluster’s superior performance on Factor 2 (Preferences)—as this suggests established ways of playing with others—and Factor 3 (Conformity)—as this result suggests an ability to follow agreed rules and acquiesce on differences of opinion. Interestingly, while these EFs are frequently viewed as cognitive tasks, they appear to be related to social skills in play. The finding that the third cluster of children, who appeared low on leadership and high on conformity in play, did not appear to do as well on EF tasks as the other two clusters (except in naming tasks), suggesting play leadership may be a crucial and indispensable ingredient for developing EFs.

Superior working memory performance in the first cluster of children suggests that moderate conformity combined with high leadership of play and salient preferences for types of play may provide more regular practice of working memory in ways that lower preferences for certain types of play may not.

The results here show the complex and probably evolving relationship between play behaviors and EF development. Questions about the differential impacts of fixed versus wider play preferences on EFs could be explored in studies specifically designed to focus on play repertoires.

### Limitations and Future Directions

The current study was preliminary in nature and leave many questions unanswered about how and why certain EF components are not associated with particular play behaviors. It is likely that certain play behaviors (e.g., leadership) are useful at certain ages of the kindergarten period, so future studies could examine relationships between behaviors across a wider age range and with greater differentiation between age groups. This may provide a finer-grained picture of the changing relationship between play and EFs. Similarly, increasing the number of items in the POBS within practicable limits may provide greater detail about the different EF components and play behaviors. In future studies, we plan to expand the sample to include children from other regions of Russia (Yakutia, Tatarstan, and Krasnodatsky Krai) in order to increase the representativeness of the sample.

It is important to test all educators separately to obtain more objective research results. Teachers may have a biased opinion about the child. If the child behaves badly due to the poor development of self-regulation, then teachers may exaggerate difficulties or underestimate his skills in play. It would be important to supplement the assessments of educators with the observation of a third-party expert on the behavior of children in the kindergarten group.

## Conclusion

This study sought to develop and validate the Play Observed Behaviors Scale (POBS) for use in large-scale studies of kindergartener’s executive functions (EFs) development. Results suggest satisfactory fit indices for three factors—play leadership, preferences and conformity—in the POBS, implying the utility of this short, 7–item scale in Russian kindergartens and potentially other similar contexts. As expected, the three factors were consistent with Vygotsky’s theoretical explanation of how children use and practice EFs in early childhood play: proposing ideas that appeal to playmates as well as the leader, having preferred types of play that reflect individual and small group interests, and being able to follow rules of the play scenario. Cluster analysis revealed that Russian kindergarteners who were high on leadership, preferences and conformity outperformed others in the sample on all EF components except visual working memory. These results broadly support the hypothesis that play behaviors reflect and may even practice EFs, and help elucidate the complex play behavior profiles of kindergarteners. However, this is preliminary work that needs to be improved.

The study is important not only from the point of view of expanding theoretical ideas about the relationship between executive functions and the child’s play behavior, but it also has a practical importance. More and more parents are resorting to the strategy of “formation of cognitive development of children.” The shown important role of the play for the development of executive functions, and children’s successful school performance in the future, will help to convincingly show parents of preschool children the need to encourage play behavior for the optimal development of their children.

## Data Availability Statement

The raw data supporting the conclusions of this article will be made available by the authors, without undue reservation.

## Ethics Statement

The study and consent procedures were approved by the Ethics Committee of Faculty of Psychology at Lomonosov Moscow State University (the approval No: 2021/72). Written informed consent to participate in this study was provided by the participants’ legal guardian/next of kin.

## Author Contributions

AV conceptualized research and contributed to results interpretation. DB introduced theoretical analyses of the research and obtained the data. OA contributed to data analyses. VS contributed to methods analyses. YC co-directed the analyses and co-authored the manuscript. All authors contributed to the article and approved the submitted version.

## Funding

This work was supported by Russian Science Foundation grant number 20-18-00423.

## Conflict of Interest

The authors declare that the research was conducted in the absence of any commercial or financial relationships that could be construed as a potential conflict of interest.

## Publisher’s Note

All claims expressed in this article are solely those of the authors and do not necessarily represent those of their affiliated organizations, or those of the publisher, the editors and the reviewers. Any product that may be evaluated in this article, or claim that may be made by its manufacturer, is not guaranteed or endorsed by the publisher.
